# Tumor-to-Tumor Metastasis: An Uncommon Case of Metastatic Breast Carcinoma to Pheochromocytoma

**DOI:** 10.30699/ijp.2025.2049764.3396

**Published:** 2025-07-01

**Authors:** Parisa Adelnia, Mitra Heidarpour

**Affiliations:** Isfahan University of Medical Sciences, Isfahan, Iran

**Keywords:** Tumor-To-Tumor Metastasis, Breast Carcinoma, Pheochromocytoma, Adrenal Metastasis, Secondary Malignancy

## Abstract

**Background & Objective:**

Tumor-to-tumor metastasis is a rare phenomenon in which one primary tumor metastasizes into another histologically distinct tumor. This report presents a unique case of breast carcinoma metastasizing to a pheochromocytoma, posing significant diagnostic and therapeutic challenges.

**Case Presentation:**

A 71-year-old woman with a history of breast carcinoma—status post mastectomy 7 years prior—presented with elevated levels of cancer antigen 15-3 (CA15-3), raising suspicion of disease recurrence or metastasis. Imaging studies revealed a mass in the adrenal gland. Surgical excision of the adrenal lesion was performed, and subsequent histopathological and immunohistochemical analyses confirmed the coexistence of two distinct tumor components: primary pheochromocytoma and metastatic breast carcinoma.

**Conclusion:**

Although tumor-to-tumor metastasis is exceedingly rare, awareness of this entity is crucial for accurate diagnosis and effective treatment planning. This case underscores the importance of considering prior malignancies in the differential diagnosis of new adrenal masses and highlights the complexities involved in managing a hormone-secreting neoplasm concurrently harboring metastatic disease.

## Introduction

Although multiple synchronous neoplasms may coexist, tumor-to-tumor metastasis (TTM) is an exceedingly rare phenomenon. TTM refers to the metastasis of one primary tumor into another histologically distinct neoplasm (1-3). Two main hypotheses attempt to explain this occurrence: the "seed and soil" theory and the "anatomical-mechanical" theory (3, 4). Breast carcinoma is known for its capacity to metastasize to various organs, yet fewer than ten cases of breast carcinoma metastasizing to pheochromocytoma have been reported in the literature. The first was described by Seitz and Schuder in 1987, and the most recent, and first in a male patient, was reported by Addo in 2023 (1, 3). This report presents another rare case of TTM from breast carcinoma to pheochromocytoma (1, 2).

## Cases description

A 71-year-old woman with a history of triple-negative breast carcinoma (ER-negative, PR-negative, HER2-negative) underwent extended radical mastectomy in 2017 followed by adjuvant chemotherapy. During a routine follow-up in 2023, an elevated serum cancer antigen 15-3 (CA15-3) level raised concern for recurrence or metastasis. A metastatic workup was initiated, including chest radiography, bone scan, brain MRI, and contrast-enhanced spiral CT of the chest and abdomen.

**Fig.1:**
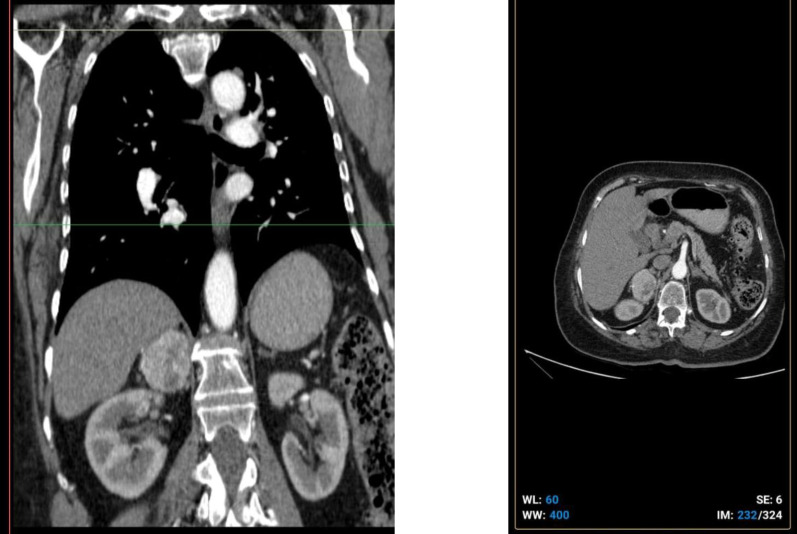
a and b: Computed tomography image showing a solid mass measuring 50 x 36 mm in the right adrenal gland.

CT imaging revealed a solid 50 × 36 mm mass in the right adrenal gland with precontrast attenuation of 27 HU and postcontrast enhancement to 90 HU—findings suggestive of metastasis given the patient's history (Fig. 1). Other imaging studies, including brain MRI and chest X-ray, showed no evidence of metastatic disease.

A core needle biopsy of the adrenal lesion was performed and histopathological analysis indicated pheochromocytoma, confirmed by immunohis-tochemical staining. Biochemical assays revealed elevated serum and urinary levels of normetanephrine and metanephrine, despite the patient being asymptomatic. Based on histological and biochemical findings, a diagnosis of pheochromocytoma was made, and adrenalectomy was carried out.

Grossly, the adrenalectomy specimen was a tan-yellow fragment measuring 5 × 4.5 × 3 cm. Serial sectioning revealed two components: a tan-to-yellow outer area with focal hemorrhage and three circumscribed white nodules, the largest measuring 0.5 cm.

**Fig.2:**
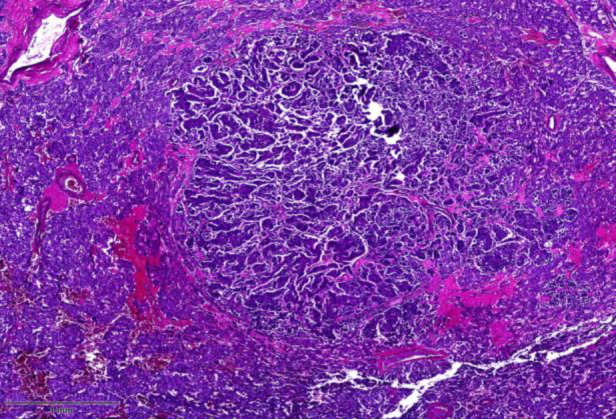
Shows the cut surface of the mass stained with hematoxylin and eosin.

Microscopic evaluation identified two histologically distinct tumor populations. The dominant component showed large polygonal cells with granular cytoplasm and uniform nuclei, arranged in trabecular and nested (zellballen) architecture typical of pheochromocytoma, with low mitotic activity and no atypical mitoses or necrosis. Within this tissue, a nodular focus of pleomorphic cells with prominent nucleoli and abundant mitotic figures was noted (Fig 2).

**Fig. 3:**
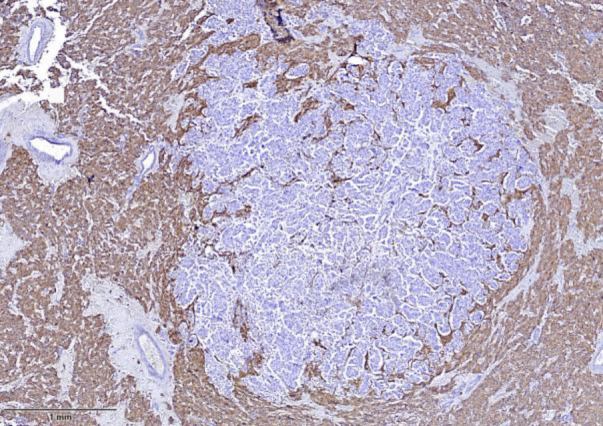
Immunohistochemistry shows reactivity of tumor cells for synaptophysin

**Fig. 4:**
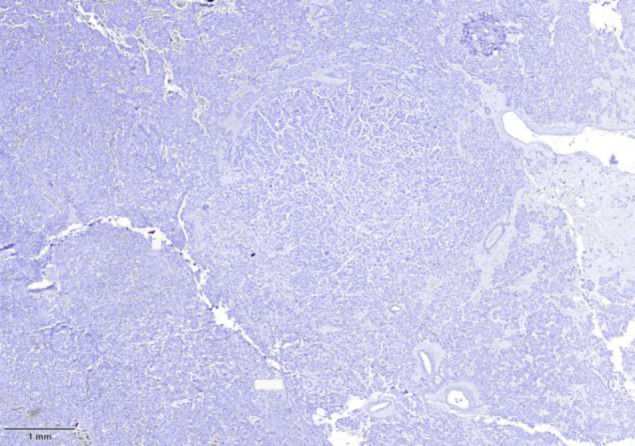
Immunohistochemistry results for ER

**Fig.5:**
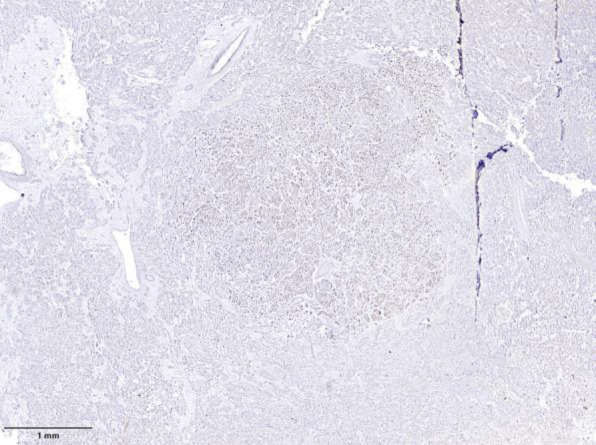
Immunohistochemistry results for GATA3

Immunohistochemistry demonstrated that the pheochromocytoma cells were positive for chromogranin A (CgA) and synaptophysin (SYN), and negative for cytokeratin (CK), GATA3, and ER (Figures 3-5). The nodular component was positive for CK, GATA3, and mammaglobin A, confirming metastatic breast carcinoma.

Following adrenalectomy, repeat serum CA15-3, metanephrine, and normetanephrine levels normalized. 

## Discussion

Tumor-to-tumor metastasis is a rare but clinically significant phenomenon that poses diagnostic and therapeutic challenges (1, 2, 4, 5). TTM should not be confused with "collision tumors," where two adjacent tumors invade each other without true metastatic involvement. The criteria for TTM include the presence of a primary tumor, metastatic infiltration that is at least partially surrounded by the benign host tumor, and exclusion of lymph nodes as recipient sites (3, 6-8).

In reported cases of breast carcinoma metastasizing to pheochromocytoma, adrenal masses are typically identified on imaging, and TTM is diagnosed only after histopathological examination (1, 5). The characteristic zellballen pattern of pheochromocytoma may be disrupted by metastatic tumor nests. Most reported cases involve invasive ductal carcinoma, and IHC panels typically include markers such as SYN and CgA for pheochromocytoma, and ER, PR, HER2, GATA3, and mammaglobin A for breast carcinoma (3, 4, 6).

Our case presented additional complexity due to the triple-negative phenotype of the primary breast tumor, which limited the utility of hormone receptor-based IHC. In such cases, a comprehensive IHC panel along with clinical history is essential for diagnosis.

The mechanism of TTM remains unclear but is believed to involve vascular, immunologic, and microenvironmental factors. Tumors like pheochromocytoma, which are highly vascular, may facilitate the entrapment and growth of circulating tumor cells (CTCs) (1, 5). Additional hypotheses include immune evasion by donor tumor cells and host tumor susceptibility due to genetic or epigenetic factors (3, 6, 9). Although triple-negative breast cancer is considered aggressive, no hereditary cancer predisposition was identified in our patient.

Adrenal involvement in breast cancer does not independently affect prognosis, which is largely dictated by the primary tumor's biology (3,7–9). Management includes adrenalectomy—particularly for symptomatic pheochromocytomas—and systemic therapy targeting the primary malignancy (4–8). Laparoscopic adrenalectomy may not be feasible for larger tumors, in which case open surgery is preferred (5). Preoperative alpha-adrenergic blockade is critical to reduce the risk of intraoperative hypertensive crises, necessitating coordinated multidisciplinary care (10).

This case illustrates the importance of maintaining diagnostic vigilance in patients with prior malignancies, even when imaging reveals unusual lesion locations (5, 11). TTM, although rare, should be considered when unexpected histological findings emerge in a known cancer patient. 

## Conclusion

This case report describes an exceptionally rare instance of tumor-to-tumor metastasis—specifically, the sixth documented case of breast carcinoma metastasizing to a pheochromocytoma (3, 4, 11). This phenomenon highlights the intricate and unpredictable nature of cancer metastasis and raises important questions about the biological mechanisms that facilitate such interactions.

Pheochromocytomas are catecholamine-producing neuroendocrine tumors of the adrenal gland (1, 5). The occurrence of metastatic breast carcinoma within a pheochromocytoma is particularly notable given the rarity of this metastatic pattern and the physiological uniqueness of the host tumor (3, 4, 6).

Documenting such rare cases contributes to a deeper understanding of tumor biology and underscores the importance of thorough histopathological evaluation in patients with a history of malignancy. Continued research into the mechanisms underlying tumor-to-tumor metastasis may improve diagnostic accuracy and inform more effective management strategies for patients with complex metastatic disease (7, 12).

## Abbreviations

Magnetic resonance imaging (MRI); CA15-3: Cancer Antigen 15-3; Contrast-enhanced spiral Computed Comography (CT); ImmunoHistoChemical (IHC); Hounsfield Units (HU); Synaptophysin (SYN); Chromogranin A(CgA); Estrogen Receptor (ER); Cytokeratin (CK); GATA Binding Protein 3 (GATA3);

Tumor-to-Tumor Metastasis (TTM). Positron emission tomography–computed tomography (PET-CT) 

## Data Availability

Data is available upon reasonable request from the corresponding author.
